# Preparation and Characterization of Whey Protein-Based Polymers Produced from Residual Dairy Streams

**DOI:** 10.3390/polym11040722

**Published:** 2019-04-19

**Authors:** Bushra Chalermthai, Wui Yarn Chan, Juan-Rodrigo Bastidas-Oyanedel, Hanifa Taher, Bradley D. Olsen, Jens Ejbye Schmidt

**Affiliations:** 1 Department of Chemical Engineering, Masdar City Campus, Khalifa University, Abu Dhabi 54224, UAE; bushra.chalermthai@ku.ac.ae (B.C.); hanifa.alblooshi@ku.ac.ae (H.T.); 2 Department of Chemical Engineering, Massachusetts Institute of Technology, Cambridge, MA 02139, USA; wychan@mit.edu (W.Y.C.); bdolsen@mit.edu (B.D.O.); 3 Department of Chemical Engineering, Biotechnology and Environmental Technology, University of Southern Denmark, 5230 Odense, Denmark

**Keywords:** protein-based polymer, copolymerization, whey protein, mechanical properties, PEGMA

## Abstract

The wide use of non-biodegradable, petroleum-based plastics raises important environmental concerns, which urges finding alternatives. In this study, an alternative way to produce polymers from a renewable source—milk proteins—was investigated with the aim of replacing polyethylene. Whey protein can be obtained from whey residual, which is a by-product in the cheese-making process. Two different sources of whey protein were tested: Whey protein isolate (WPI) containing 91% protein concentration and whey protein concentrate (WPC) containing 77% protein concentration. These were methacrylated, followed by free radical polymerization with co-polymer poly(ethylene glycol) methyl ether methacrylate (PEGMA) to obtain polymer sheets. Different protein concentrations in water (11–14 *w*/*v*%), at two protein/PEGMA mass-ratios, 20:80 and 30:70, were tested. The polymers made from WPI and WPC at a higher protein/PEGMA ratio of 30:70 had significantly better tensile strength than the one with lower protein content, by about 1–2 MPa (the best 30:70 sample exhibited 3.8 ± 0.2 MPa and the best 20:80 sample exhibited 1.9 ± 0.4 MPa). This indicates that the ratio between the hard (protein) and soft (copolymer PEGMA) domains induce significant changes to the tensile strengths of the polymer sheets. Thermally, the WPI-based polymer samples are stable up to 277.8 ± 6.2 °C and the WPC-based samples are stable up to 273.0 ± 3.4 °C.

## 1. Introduction

Raw materials from renewable and agricultural sources have been proposed to produce plastics for food packaging and shopping bags for many years [1,2]. With increasing environmental concerns surrounding non-biodegradable plastics, several options have been explored to replace such conventional plastics. Since some of the renewable materials, which are mainly plant-based, are first generation feedstocks, which compete with the food security, utilizing second-generation feedstocks (biomaterials which are by-products, residues, or wastes from other processes) is the preferable option. One such option is the production of plastics from whey proteins, which can be obtained from the by-product/residues of cheese-making processes and expired dairy products.

Cow and goat milk mainly consist of 80% casein proteins and 20% whey proteins [3]. Casein is a major component of cheese [4], i.e., in cheese production, casein is coagulated, while the residual whey is normally discarded. Whey proteins, also known as serum proteins [4], consist of several globular proteins. The most abundant protein in whey is beta-lactoglobulin, making about 50% of the total whey protein, while the second-most abundant is the alpha-lactalbumin, making up about 20% of the whey protein [5]. The remaining fractions are minor protein/peptide components (e.g., immunoglobulins, lactoferrin, lactoperoxidase, serum albumin, lysozyme, and growth factors) [5].

Whey, a by-product of the cheese industry can be used for a wide variety of applications: for example, as a food supplement and in protein drinks [6]. Generally, in order to make 1 kg of cheese, about 9 L of whey are generated [7], of which approximately 0.55% of it is whey protein [8]. The production of whey in the world is about 160 million tonnes in 2008, which has risen to 200 million tonnes in 2011, thereafter with an increase rate of 2% per year [5,9]. These whey wastes were also discarded into waterways, causing environmental problems [10]. Today, protein is extracted from the waste whey and valorized for applications in food and pharmaceutical industries, among others [6,11,12]. Of the produced whey in the United States, 65% is utilized further for human consumption, and 35% for animal feed [13]. In recent years it is not well documented how much product from the dairy industry is recycled and how much is discharged [13]. In the UAE, large amount of fresh milk expires and is discharged daily. For example, in a Dubai dairy farm, 7 tonnes of expired milk is wasted daily [14]. The whey protein from expired dairy products therefore have a high potential for producing protein-based plastics. 

Different studies have investigated the use of proteins to form polymeric materials primarily for packaging purposes. Milk proteins have also been used to produce plastics, especially for food packaging. Films and coatings for food products, which are also edible, have been produced successfully from milk proteins [15]. Ramos et al. [16] studied the effect of whey protein purity and glycerol (plasticizer) content on the physical properties of edible films made from them. It was found that WPI films exhibited higher mechanical resistance, elasticity, and transparency values, but lower moisture content, film solubility, water vapor permeability, and color change values than their WPC counterparts, for the same content of glycerol. Plastic films from milk proteins were also created for specific application, such as for cheddar cheese packaging, from the study done by Wagh et al. [17]. Casein and whey proteins were plasticized with glycerol and sorbitol, and the oxygen and water vapor barrier and tensile properties were determined. The tensile strengths and elongation of WPC plasticized with glycerol were generally greater than those with sorbitol, while the elastic moduli were somewhat equivalent [17]. Jones et al. [18] investigated the thermal, viscoelastic and antibacterial properties of albumin, soy, and whey bioplastics using three types of plasticizers: water, glycerol, and natural rubber latex (NRL). While the whey protein film plasticized with glycerol showed the highest tensile strength, the film plasticized with NRL has the highest modulus and the film plasticized with water the greatest elongation-at-break. 

Besides milk proteins, proteins from different sources including plant (corn zein, wheat gluten, soy proteins) and animal proteins (collagen, gelatin, keratin and myofibrillar) have been explored for polymer production. Cuq et al. [1] have extensively reviewed different types of plant and animal proteins that could be used to produce agricultural polymers which are used for packaging. A paper by Flieger et al. (2003) [2] reviews the biodegradable plastics that are made from different renewable sources, including those from microbial fermentations such as polyesters and neutral polysaccharides, and those prepared from chemically-modified natural products such as starch, cellulose, chitin, or soy proteins. Soy protein is found to be one of the most common proteins that are used to produce biodegradable plastics [2,19,20]. 

There are several methods in which proteins can be copolymerized with other monomers to produce plastic sheets or films. One such method is through free-radical polymerization, where the side chains and N-termini in the protein molecules can conjugate with other compounds to form polymer, after the protein structure has been modified by functionalizing it with a reactive group, e.g., methacrylating it with methacrylic anhydride, which is well-established in the field of synthesis of GelMA or gelatin methacrylate [21,22]. The modified protein is then copolymerized with a monomer to form polymeric or plastic-like materials. Several factors are hypothesized to play an important role in formation of biomaterials and their final properties: protein formulation, polymerization stoichiometry, and the processing technique [15,23,24,25]. Protein formulation represents the quantitative and qualitative properties of bulk protein fraction obtained from the dairy products. Protein purity, length of peptide chains (which can be controlled by hydrolysis), protein denaturation, and most certainly degree of methacrylation are some of the factors that can influence properties of the final materials. Polymerization stoichiometry refers to the mass ratio between the soft-segment building comonomer and hard-segment building proteins. The mechanical behavior of the elastomer is highly affected by this proportion. The third group, the processing parameters, is related to reaction conditions (heat or chemical catalysis), downstream processing (drying, molding, etc), and plasticizers used [15,23]. 

In terms of economics, the purity of the protein, as well as its concentration in water can impact the cost of the final products. Commercial whey protein concentrates (WPC) are produced by fractionation with ultrafiltration (UF) and diafiltration (DF) methods, to remove lactose, minerals, and other low-molecular weight components, resulting in WPC with ≥75% protein [26]. Whey protein isolates (WPI), on the other hand, are manufactured by stirred-bed ion exchange adsorption process, where the pH of the proteins is adjusted and the proteins are eluted from the ion exchanger, concentrated by UF and spray dried, resulting in WPI with ≥90% protein [26]. The commercial value of the products is determined by the purity. The higher value intuitively requires more expensive modules, types of membrane, and even methods to control fouling [4]. Therefore, the use of either WPC or WPI would definitely impact the cost of the process and the final products. Moreover, by using less protein (greater dilution in water), the cost may also be minimized. Hence, the purity and the concentration of proteins in the starting materials to produce polymers are also important factors, as far as the economy of the process is concerned. 

The method that is followed in this paper is based on the research done by Chan et al. [24]. This study goes beyond that work, optimizing different polymerization proportions with the copolymer poly(ethylene glycol) methyl ether methacrylate (PEGMA) and comparing the two different types of whey proteins: whey protein concentrate (WPC) and whey protein isolate (WPI). The objectives of the present manuscript are: (1) To compare the mechanical and thermal properties of the polymers obtained from WPI and WPC (2) to compare the mechanical properties of different protein-to-monomer proportions (namely 20:80 and 30:70), and (3) to analyze the mechanical properties of the different variations (11%–14%) of protein concentration in water, given a fixed dry weight concentration (20% and 30%).

## 2. Materials and Methods 

### 2.1. Materials 

Whey protein concentrate (WPC) that contains 77% protein, as per supplier information, was purchased from the supplier *Natur-Drogeriet* based in Hørning, Denmark, while whey protein isolate (WPI) that contains 91% protein, was purchased from the BiPro company (Eden Prairie, Minnesota, USA). These two protein sources were chosen as the model crude protein mixture as they are well-studied and widely available agricultural-based proteins, obtained mainly from the dairy industry. Poly(ethylene glycol) methyl ether methacrylate (PEGMA, M_n_ = 500 g·mol^−1^) was used as the co-polymerization monomer and was purchased from Sigma-Aldrich (St. Louis, Missouri, USA). Methacrylic anhydride, *tetra*-methylethylenediamine (TEMED), and ammonium persulfate (APS) were also purchased from Sigma-Aldrich (St. Louis, Missouri, USA).

### 2.2. Methacrylation and Polymerization Reactions

Polymerizable whey protein was prepared through an amine-based reaction with methacrylic anhydride to introduce (meth)acryloyl moieties onto milk-derived proteins. It is important to make sure that the reactions are not carried out in acidic conditions since the proteins can precipitate at pH around 5. At higher pH conditions, the amine groups on lysine side chains and the protein N-termini can react with the anhydride in the methacrylation step.

Prior to the process of methacrylation, the pH of the protein solution is increased to above a neutral level of pH 7. This is mainly to prevent proteins precipitation when methacrylic acid forms during the reaction and the pH drops. Proteins precipitation occur at the isoelectric point (pH about 4–6), which must be avoided before polymerization. 

**Figure 1:**
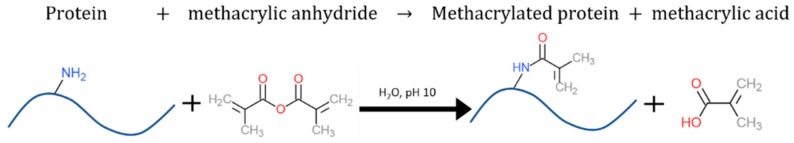
Methacrylation reaction of protein (adapted from Chan et al. [24]).

Methacrylation was used to chemically activate the selected proteins before their further co-polymerization with PEGMA. Methacrylation is a reaction that occurs randomly on different amine groups along the polypeptide chain, more specifically on the ε-amines of lysines and the N-terminal amine of the protein chain [24]. Figure 1 presents a diagram of the protein methacrylation reaction. Methacrylic anhydride was added, at a dosage of 50 µL/g_protein, to the WPC or WPI solutions, in a 20 mL reaction vessel, and incubated for 18 h at a temperature controlled Innova42 incubator shaker (New Brunswick Scientific, Göteborg, Sweden) at 25 °C and 100 rpm. The resulting solution contains methacrylic acid and the selected methacrylated proteins.

**Figure 2:**
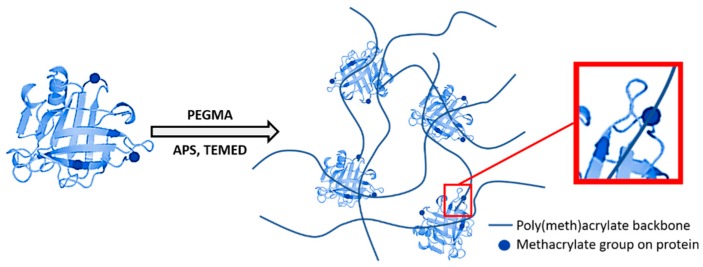
Copolymerization of the methacrylated protein with PEGMA in presence of initiator APS and catalyst TEMED (adapted from Chan et al. [24]).

The resulting methacrylated protein was co-polymerized with PEGMA to form polyethylene-like plastics, at two protein/PEGMA mass ratios: 20:80 and 30:70. A 20% *w*/*v* solution of ammonium persulfate (APS) was added as an initiator at a concentration of 4% *v*/*v* w.r.t. PEGMA. TEMED was used as a catalyst at a concentration of 0.2% *v*/*v* w.r.t. PEGMA. Figure 2 illustrates the reaction carried out to produce the polymer. The resulting mixture was then placed (by pipette) in between two parallel glass plates with a spacer of 1 mm and left at ambient conditions (25 °C) for 2 h to form gel-like sheets. The sheets were then peeled-off and dried at 60 °C for 46 h to form elastomers of thickness of about 0.5 mm. The resulting sheets were maintained at 55 ± 5% relative humidity for a minimum of 2 days before the tensile and thermal analyses. Additional to the two protein/PEGMA ratios (20:80 and 30:70), as mentioned above, 4 protein concentrations in water, 11–14% *w*/*w* were tested, for both of the selected proteins WPI and WPC. This resulted in a total of 16 unique polymer sheets, which were tested for their tensile and thermal characteristics. 

### 2.3. Mechanical and Thermal Analysis 

The resulting sheets were cut into 50 × 10 mm rectangular strips with a gauge length of 30 mm. 3 strips were obtained from each sheet. Tests were carried out using a universal tensile testing machine (Zwick Roel Z005, Ulm, Germany) with 20 N load cell and at 1 mm/min strain rate. The thickness of the strips was measured with a digital Vernier caliper (detection limit = 0.01 mm). The thickness of the rectangular specimens used for tensile testing was about 0.45 ± 0.15 mm. 

The thermal stability of the resulting polymer sheets was analyzed using a NETZSCH High Temperature TGA-*STA449F3-Jupiter* thermogravimetric analyzer (TGA, NETZSCH-Gerätebau GmbH, Selb, Germany), under inert atmosphere (nitrogen gas) at a heating rate of 10 °C/min from 30 °C to 800 °C using approximately 10 mg of samples.

### 2.4. Statistical Analysis

For each specimen, three representative samples data were collected, and average values were considered. Differences between samples were deliberated as significant by considering 95% confidence level. All results obtained are expressed as mean ± margin of error at a confidence level of 95%.

## 3. Results and Discussion

**Figure 3:**
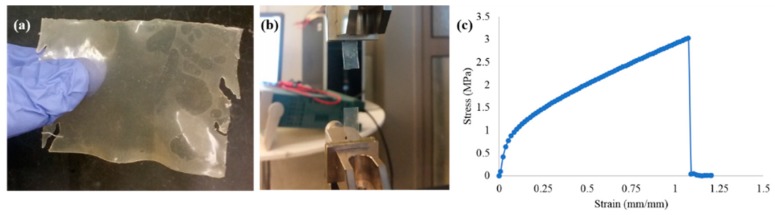
(**a**) Sample plastic sheet post drying, (**b**) Sample plastic sheet breakage at the ultimate tensile strength, (**c**) Stress-strain curve of a sample.

**Figure 4:**
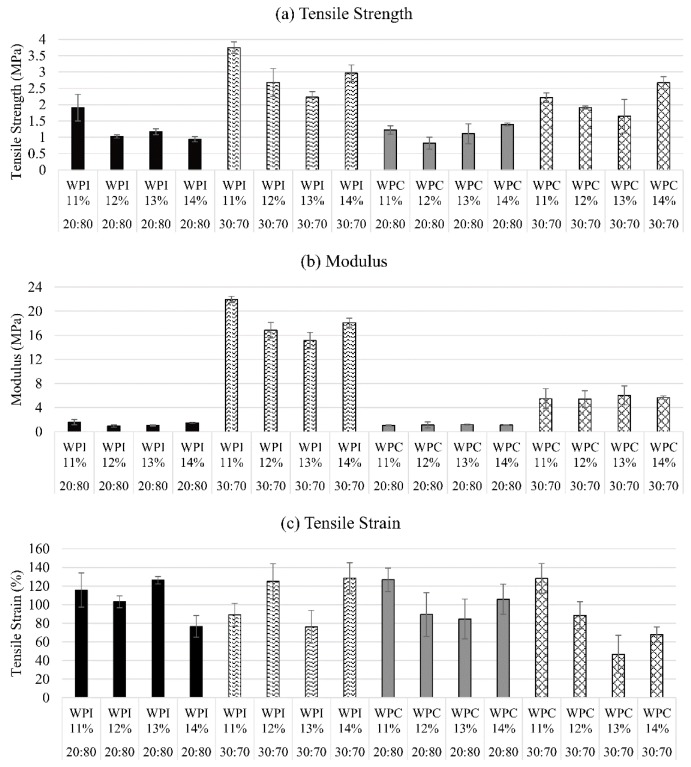
Average (**a**) tensile strength (**b**) modulus and (**c**) tensile strain, of all polymer samples, all with error bars at 95% confidence level. Each group of bars color/pattern is a set of 4 different protein concentrations in water from 11–14 *w*/*v*%.

A sample plastic sheet post-drying, along with its specimen under tensile test and its stress vs. strain curve are as shown in Figure 3. The *tensile strength*, *σ* (MPa), *modulus of elasticity*, *E* (MPa), and the *tensile strain (elongation or extension-at-break)*, *ε* (%) results obtained as mean ± margin of error at a confidence level of 95% are shown in Figure 4. 

### 3.1. Effect of Protein/PEGMA Ratio 

**Figure 5:**
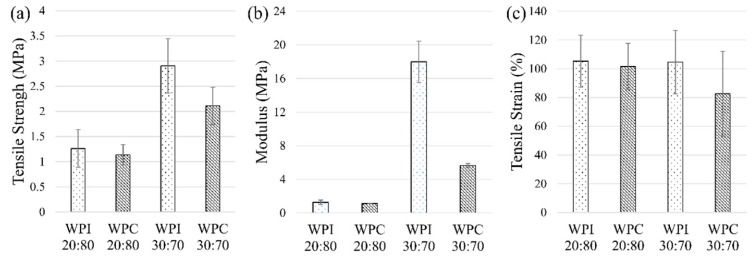
20:80 vs. 30:70: Average (**a**) tensile strength (**b**) modulus and (**c**) tensile strain, all with error bars at 95% confidence level.

One of the objectives was to observe the difference when varying the amount of the hard domain (protein) to the soft domain (copolymer PEGMA). This study shows that the samples for the WPI and WPC at 20:80 protein/PEGMA ratio were softer in texture than the samples obtained from those at the 30:70 ratio. The average tensile strength, modulus, and tensile strain of the WPI and WPC polymers at the different ratios of protein/PEGMA, with error bars at 95% confidence level are as shown in Figure 5. The values obtained in Figure 5 are the averages of all the different protein in water concentration, for the respective type of protein (either WPI or WPC), at the given protein/PEGMA ratio.

The polymer samples with protein/PEGMA ratio of 20:80 exhibit lower tensile strength compared to that of the protein/PEGMA ratio of 30:70 (Figure 5a). The samples with a protein/PEGMA ratio of 30:70 exhibit greater tensile strength, the average value of up to 2.9 ± 0.5 MPa for the WPI samples and 2.1 ± 0.4 MPa for the WPC samples. There is a clear significant difference between the two polymer composition types at the 95% confidence level. This is also evident in the modulus results. The WPI samples with protein/PEGMA ratio of 30:70 exhibit much greater modulus, at 18.0 ± 5.6 MPa, compared to its 20:80 counterpart which has a modulus of 1.3 ± 0.3 MPa (Figure 5b), a significant difference of over 10 MPa. For the WPC samples, the difference between the 20:80 and 30:70 protein/PEGMA (1.1 ± 0.0 MPa and 5.6 ± 0.2 MPa, respectively) is about 4 MPa, which is also significant at the 95% confidence level. By increasing the amount of the hard domain (whey protein) in the concentration, the polymer sheets yield better tensile strength by about 1–2 MPa and the modulus by over 10 MPa for WPI and 4 MPa for WPC samples. This implies that if the amount of whey protein is increased in this dry weight concentration, a better-performing plastic sheet can be obtained. However, it was found from this study that if the protein/PEGMA ratio was greater than 30:70 (i.e., at 40:60), the polymer sheets were more brittle after oven drying and could not be used for tensile strength testing. On the other hand, if the protein/PEGMA ratio was lower than 20:80 (i.e., at 10:90), then the polymer sheets would be too fragile to handle, i.e., they would be too soft in texture and would tear off easily, even before oven drying, which could not be mechanically tested. Hence, the optimal concentration in terms of tensile strength in this study is the polymers with protein/PEGMA ratio of 30:70. The tensile strain property, however, showed no difference among the polymer sheets obtained from different protein/PEGMA ratios (Figure 5c).

### 3.2. Effect of Protein in Water Concentrations 

The results for each unique sample shown in Figure 4 were used to analyze the variations of the different concentrations of protein dissolved in water. As shown in Figure 4a, the ultimate tensile strength, i.e., the maximum stress the polymer can withstand before the fracture, of the WPI 11% (30:70) is the highest (3.8 ± 0.2 MPa), among all the polymers tested. WPI 11% (20:80), compared among its 20:80 protein/PEGMA counterparts, has the highest tensile strength (1.9 ± 0.4 MPa). No statistical difference can be observed among the WPI 12%, 13%, and 14%, when compared among the same group of protein/PEGMA proportion. The WPC polymer sheets, on the other hand, showed no statistically significant variations of the tensile strengths among the different concentrations of protein in water (11–14%), except for the WPC 14% (30:70) that has the highest tensile strength at 2.7 ± 0.2 MPa, when compared to its fellow WPC samples. However, this is not statistically different from the WPI 30:70 samples of the concentrations 12%, 13%, and 14%. Thus, except for the WPI 11% (30:70), there are no significant variations among the different protein concentrations in water with regards to their tensile strength. The reason for the WPI 11% (30:70) performing best (3.8 ± 0.2 MPa) may be attributed to the fact that it exhibits stiffer structure due to the hard domains imparted from the proteins, but not too stiff that they can be more prone to breakage at a higher protein concentration in water (12%+). At greater than 14%, the polymer sheets obtained were too brittle after oven drying and could not be mechanically tested, whereas those below 11% were too fragile to handle, and tore very easily as the sheets were peeled off even before oven drying. 

Figure 4b shows the average modulus of all the polymer samples. Similar to the tensile strength result, the WPI 11% (30:70) exhibited the highest modulus among all of the polymer samples tested, at 21.9 ± 0.5 MPa. This is significantly greater than the modulus of the all the other samples. There are no significant differences among the other concentrations 12–14 *w*/*v* % of the WPI (30:70) samples. Similarly, all the other groups of polymers have no significant differences among their different protein concentrations in water at 11–14 *w*/*v*%. 

It is important to note that while the other group of polymers exhibited similar modulus to the tensile strength results, the group of WPI (20:80) samples exhibited moduli of about 5–10 times greater than their tensile strengths in MPa. This is already evident from the results shown in Figure 5, but Figure 4 also proved this point with the results displayed as each unique polymer samples’ results. The modulus (MPa) results for each unique sample also show that the 30:70 set of polymers exhibited 5–20 times the modulus of their 20:80 counterparts. The tensile strengths might not have given a clear idea of the material strength of the WPI (30:70) set of polymers, but the modulus results made this conspicuous. Although the WPC (30:70) set of polymers also have high modulus (4–8 MPa), these are still significantly lower than the ones of the WPI (30:70) set.

The tensile strain (elongation or extension-at-break) of the WPI and WPC samples is shown in Figure 4c. As can be observed, there is no difference in the tensile strain among the different WPI and WPC samples at different protein concentrations in water of 11–14%. 

This study shows that the tensile strengths of the produced polymers are in the range of 1–4 MPa. This is similar to the tensile strength of 0.7–4 MPa of the milk protein films that have been used for cheese packaging [17]. The elastic modulus of these whey protein films plasticized with glycerol and sorbitol ranged from 2–5 MPa [17], which is lower than our polymer films with 30:70 protein/PEGMA concentration (5–22 MPa), but higher than our polymer films with 20:80 protein/PEGMA concentration (1–2 MPa). Our produced polymers also have slightly better tensile strengths but lower moduli than the plasticized WPI films embedded with porous silica (SiO_2_) coated titanium (TiO_2_), which have tensile strength of about 1–2 MPa and modulus of 20–60 MPa [27]. Compared to other polymerized milk protein films such as those obtained from blends of whey protein isolate and glycerol having tensile strength ranging from 1.08 to 29.1 MPa [15], the polymer sheets obtained in this study could be further improved. Compared to synthetic polyethylene films such as LDPE (low density polyethylene) (tensile strength = 9.0–18.0 MPa; modulus = 0.15–0.29 GPa) [28] and HDPE (high density polyethylene) (tensile strength = 15.2–45.0 MPa; modulus = 0.62–1.45 GPa) [29], the polymer sheets produced here have much lower tensile strength and modulus. The mechanical property of any copolymerized protein film depends on parameters such as the plasticizers or comonomers used, the cross-linking agents, the chemical and physical conditions of experimental procedure, and the texture of the polymer sheet. The film flexibility can be increased by increasing the amount of plasticizer or comonomer as this helps in weakening the intermolecular forces between adjacent polymer chains [25], which can also result in softer texture and lower tensile strength. On the other hand, proteins that are unplasticized, just like other common dry proteins, are very brittle [24]. Hence, an appropriate blend between the soft rubbery domain and the hard domain to achieve the best possible tensile strength with proper stiffness and flexibility will need to be further investigated with other plasticizers.

### 3.3. Thermal Stability Results 

**Figure 6:**
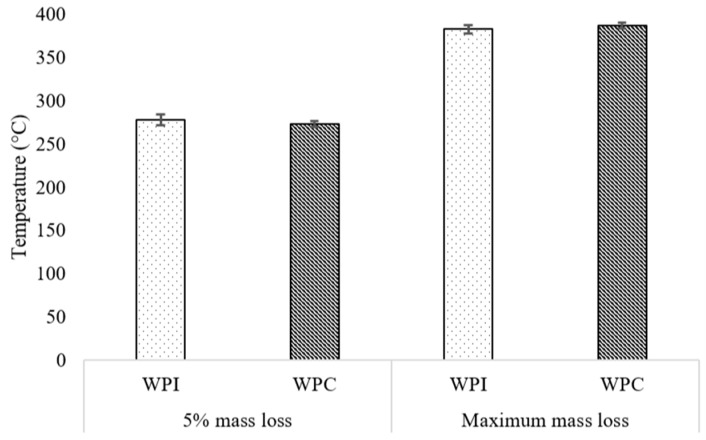
Five percent mass loss temperatures (T_5%_) and maximum mass loss temperatures (DTG_max_) of WPI and WPC-based polymer samples.

**Figure 7:**
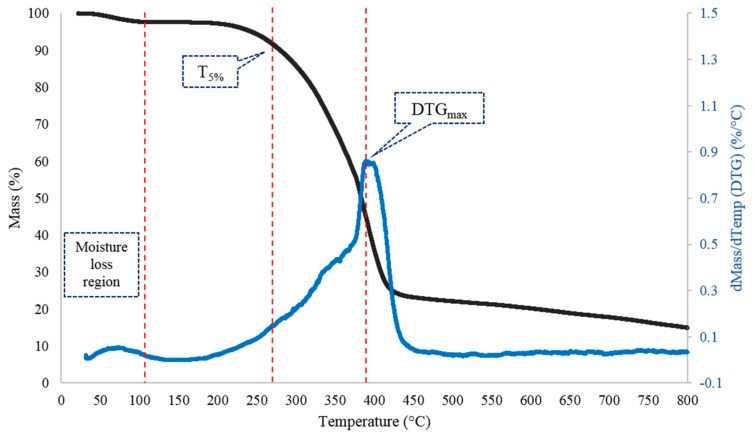
Mass loss curve (black curve) with its first derivative (blue curve) of a representative polymer sample. Moisture loss occurs until about 110 °C. After that, the polymer sample is thermally stable up to about 275 °C (where 5% mass loss occurs post-moisture loss) and the maximum mass loss is observed at the peak of the derivative, i.e., about 390 °C.

Thermogravimetric analysis (TGA) test was performed on each unique polymer sample (at all protein-in-water concentrations and protein/PEGMA ratios) and used to determine the 5% and maximum mass loss decomposition temperatures (T_5%_ and DTG_max_, respectively). The thermal stability of the polymer samples is determined by the temperature at which about 5% mass loss occurs (T_5%_), and the results show that the polymer samples are all thermally stable up to about 275 °C. At a confidence level of 95%, the thermal stability is in the range of 277.8 ± 6.2 °C for WPI-based polymer samples and 273.0 ± 3.4 °C for WPC-based polymer samples (Figure 6). Meanwhile, the temperature range where the polymer starts disintegrating and the large molecular weight bonds break occur where the maximum mass loss is observed, i.e., about 60% mass loss (DTG_max_). At a confidence level of 95%, the temperatures at which the maximum mass loss occurs are 382.8 ± 4.7 °C for WPI-based polymer samples and 387.1 ± 3.7 °C for WPC-based polymer samples (Figure 6). A typical mass loss and its derivative curve of a representative polymer sample is as shown in Figure 7. The moisture loss region, the point of T_5%_ and DTG_max_ are annotated in the figure. There is no significant difference in the thermal stability among all the polymer samples at all concentrations of protein-in-water and protein/PEGMA ratios tested. Below 110 °C, small mass losses (<5%) may be observed, and this is due to the evaporation of water from the samples.

The thermal property of the polymer samples observed here is similar to that of the polymer sheets produced using whey protein cross-linked with another copolymer, PHPA (poly-(hydroxypropyl acrylate)), where they remain thermally stable up to 300 °C and has the maximum mass loss at about 400 °C [24]. In another study, the WPI films embedded with porous silica (SiO_2_) coated titania (TiO_2_) were thermally stable up to about 200 °C [27], which is lower compared to the WPI and WPC-based plastic sheets produced in this study. Plastics made from other proteins such as soy proteins were stable up to 300 °C [30]. On the other hand, other conventional polymers such as HDPE have thermal stability up to about 420 °C–567 °C and that of LDPE in the range of 386 °C—577 °C [31], which are higher than that of the plastic sheets produced in this study. Hence, the polymer sheets obtained in this study have thermal stability lower than that of conventional plastics, but are similar to that of other bio-based plastics.

## 4. Conclusions

The synthesis of protein-based thermoset elastomers was carried out with whey protein isolate (WPI) and whey protein concentrate (WPC). Both of them show considerable potential for use as polymers or plastics through free-radical polymerization with a copolymer that has a low glass transition temperature like PEGMA. Since WPI samples require more processes in the fractionation of the protein, the WPC may be a more cost-effective input that produces equally performing plastic sheets. However, by comparing the different proportions of protein, which act as a hard domain, to the copolymer PEGMA, which acts as the soft block, the mechanical property of the plastic sheets can vary considerably. The polymer samples that are produced from a lower amount of the hard domain (whey protein) that has a protein/PEGMA proportion of 20:80 exhibited softer texture than the one that has a higher proportion of 30:70. However, the polymer sheets with 30:70 protein:copolymer proportion yield better tensile strength by about 1–2 MPa and modulus by about 5–20 MPa. Meanwhile, variations of the protein concentration solution in water (11–14%) showed no significant differences in their tensile properties. Since the cost is higher at higher concentration of protein, it may be more cost-effective to use less amount of protein in the starting material. The thermal stability of all the polymer samples tested were generally in the range of 275 °C, which is comparable to other plastics. Since the tensile strengths (1–4 MPa) of the polymer samples are still somewhat low, improvements will have to be made before they may be used for packaging purposes. The variations of the hard protein domains to the soft copolymer blocks may be increased, say, testing the samples at 25:75 or 35:65 protein/PEGMA ratios maybe considered. By using more variations, the optimal composition that results in better mechanical strengths of polymer sheets may be obtained. Once the tensile strengths of the polymer samples are improved to above 4 MPa, then there is a potential in using these plastics for packaging, which can replace the use of other conventional plastics in the future.
